# Enhanced Horizontal Transfer of Antibiotic Resistance Genes in Freshwater Microcosms Induced by an Ionic Liquid

**DOI:** 10.1371/journal.pone.0126784

**Published:** 2015-05-07

**Authors:** Qing Wang, Daqing Mao, Quanhua Mu, Yi Luo

**Affiliations:** 1 College of Environmental Science and Engineering, Ministry of Education Key Laboratory of Pollution Processes and Environmental Criteria, Nankai University, Tianjin 300071, China; 2 School of Environmental Science and Engineering, Tianjin University, Tianjin 300072, China; Catalan Institute for Water Research (ICRA), SPAIN

## Abstract

The spread and propagation of antibiotic resistance genes (ARGs) is a worldwide public health concern. Ionic liquids (ILs), considered as “environmentally friendly” replacements for industrial organic solvents, have been widely applied in modern industry. However, few data have been collected regarding the potential ecological and environmental risks of ILs, which are important for preparing for their potential discharge into the environment. In this paper, the IL 1-butyl-3-methylimidazolium hexafluorophosphate ([BMIm][PF6]) (0.001-5.0 g/L) was tested for its effects on facilitating ARGs horizontal transfer mediated by plasmid RP4 in freshwater microcosms. In the horizontal transfer microcosms, the transfer frequency of plasmid RP4 was significantly enhanced (60-fold higher than untreated groups) by the IL [BMIm][PF6] (1.0 g/L). Meanwhile, two strains of opportunistic pathogen *Acinetobacter* spp. and *Salmonella* spp. were isolated among the transconjugants, illustrating plasmid RP4 mediated horizontal transfer of ARGs occurred in pathogen. This could increase the risk of ARGs dissemination to human pathogens and pose great threat to public health. The cause that [BMIm[PF6] enhanced the transfer frequency of plasmid RP4 was proposed by suppressed cell membrane barrier and enhanced cell membrane permeability, which was evidenced by flow cytometry (FCM). This is the first report that some ILs facilitate horizontal transfer of plasmid RP4 which is widely distributed in the environment and thus add the adverse effects of the environmental risk of ILs.

## Introduction

The emergence and persistence of clinically relevant antibiotic resistance genes (ARGs) in natural and engineered environments has become a major global health issue [1–4]. Antibiotic resistance in bacteria can be intrinsic or acquired by antibiotic selective pressure [5–7]. Acquired resistance is the main reason for ARGs proliferation and it occurs between individual cells or species mainly by horizontal transfer processes [8–10]. The primary mechanisms of horizontal transfer are conjugation (plasmids are transferred from a donor cell to a recipient cell), transformation (uptake of naked DNA), and transduction (bacteriophages as transporters of genetic information) [11–13]. Conjugation is considered the principal mode for antibiotic resistance transfer, because many resistance genes are situated on mobile elements, such as plasmids [14,15], integrons [16–18] and transposons [9,19]. Meanwhile, environmental selective pressure other than antibiotics, such as metals [20,21], detergents [22], and even nanomaterials [23], can facilitate the transmission of ARGs by horizontal transfer.

The critical role played by plasmids in the horizontal transfer of ARGs has been widely recognized and is particularly salient [12, 24, 25]. Plasmid-mediated horizontal transfer of resistance plasmids to indigenous bacteria takes place in soil [26] and wastewater environments [27]. Dissemination and propagation of resistance genes could occur via horizontal transfer from resistant bacteria to susceptible strains either between different species or across genera [19]. In particular, *in situ* transfer of self-transmissible plasmid RP4 has been reported between *Xanthomonas campestris* and *Erwinia herbicola* [28], and from *Escherichia coli* to indigenous bacteria in soil [29], activated sludge [30] and seawater [31]. This 60-kb broad-host-range conjugative plasmid of the typical incompatibility group P1 (IncP) harbors multi-resistance genes for ampicillin resistance (Ap^R^, *tnp*R gene), kanamycin resistance (Km^R^, *aph*A gene), and tetracycline resistance (Tc^R^, *tet*A gene) [32].

Ionic liquids (ILs) are organic salts with high polarity and ionic conductivity, a wide electrochemical window, and excellent chemical and thermal stability. They are considered “environmentally friendly” replacements for industrial volatile organic compounds [33, 34]. As novel materials, many studies have highlighted ILs as representing a state-of-the-art, innovative approach to sustainable chemistry, using the argument that the vapor pressure of the compounds is immeasurably low and they are not flammable [33,35]. The most common applications of ionic liquids appear regularly in various disciplines, e. g., energy [36], biotechnology [37,38], chemistry [39,40], chemical engineering [34,41], polymer materials [42,43], and nanotechnology [44,45].

There has not yet been any detection report of ILs in the environment, however, to prepare for the possible environmental release of ILs, recent efforts have been toward carefully evaluating their toxicity, fate, and potential environmental effects [46,47]. In fact, many ILs are toxic to microorganisms as a result of increased osmotic pressure, effects on unique membrane porins, and inhibition of enzymatic activity [48, 49]. ILs with imidazolium have critical inhibitory effects on the growth of a variety of bacteria and fungi [50–52] as well as higher antimicrobial activity with increasing alkyl chain length [53, 54]. Ganske and Bornscheuer [48] found low concentration (<1.5 g/L) of the IL (1-butyl-3-methyl imidazolium tetrafluoroborate, [BMIM][BF4]) had no effect on the *Escherichia coli*, *Pichia pastoris*, *Bacillus cereus* and inhibited growth with 10 g/L of [BMIM][BF4]. These effects have been attributed to the structural similarity between some ILs and biocides, detergents and antibiotics [53, 55]. Interestingly, alteration of the cell membrane composition in an *Enterobacter lignolyticus* strain makes the bacterium resistant to the IL (1-ethyl-3-methylimidazolium chloride, [C2mim][Cl]) attributable to increasing cyclopropane fatty acids in the cell membrane. However, whether ILs have the potential to facilitate plasmid horizontal transfer and therefore promote the transmission and proliferation of ARGs among environmental bacteria remains unclear.

In this study, an IL 1-butyl-3-methylimidazolium hexafluorophosphate ([BMIm][PF6]) (0.001–5.0 g/L) was tested for its effects on horizontal transfer of resistance genes mediated by plasmid RP4 in freshwater microcosms. The horizontal transfer of plasmid RP4 were tracked from donor strain (*E*.*coli* K12) to recipients strain (indigenous bacteria in microcosm) treated with [BMIm][PF6]. Furthermore, alteration of cell membrane permeability by flow cytometry (FCM) was measured to explore a possible mechanism by which the [BMIm][PF6] facilitates horizontal transfer of plasmid RP4. This is the first study showing the IL-induced promotion of horizontal transfer of ARGs mediated by plasmid RP4 in a freshwater environment.

## Materials and Methods

### Setup of plasmid RP4 horizontal transfer in microcosm

Setup of plasmid RP4 horizontal transfer in microcosms was based on the OECD 308 test [56] to test the effects of the IL [BMIm][PF6] on the plasmid RP4 horizontal transfer to indigenous bacteria in the microcosm. The freshwater sample was collected from the Water Parke (39°6’13”N, 117°9’21”E) in Tianjin, China on Sep 2013. No specific permission was required as this is a public park and is open to visit and scientific research. In addition, the authors confirm that no endangered or protected species were involved in this study. Water properties are described in S1 Table and detection method of water samples in SI-5 in S1 Text. The strain of rifampicin resistance (Rif^R^) *E*.*coli* K12 (ATCC 47076) harboring the plasmid RP4, carrying ampicillin, kanamycin and tetracycline resistance (Ap^R^, Km^R^, and Tc^R^), was used as the RP4 donor strain. The recipients were indigenous bacteria in freshwater microcosms. Meanwhile, in microcosms, recipient isolates were negative in the Rif^R^ screening and PCR screening, *tra*F gene (indicator for RP4) and *aph*A gene (kanamycin-resistance gene on RP4) were negative also in the PCR screening.

The water samples (1800 mL in 2000 mL break) were stabilized for 3 hours at 4°C in a refrigerator to remove sediment. Supernatant (1500 mL) was collected and supplemented with 1% (V:V) Luria-Bertani (LB) media, incubated overnight on a shaker incubator (160 rpm) at 30°C, and diluted to reach microbial concentrations (the optical densities) of OD_600_ = 0.400. Water samples (200 mL) were then supplemented with 1% (V:V) *E*.*coli* K12 donor strains (approximately plasmid RP4 concentration of 5 μg/mL), making microcosms. Donor-free microcosms also used as controls (no horizontal transfer in donor-free microcosms in pre-experiment). The microcosms were spiked with an IL [BMIm][PF6] (>99% pure, Chinese Academy of Science), at initial concentrations of 0, 0.001, 0.01, 0.1, 1.0, and 5.0 g/L. Each concentration was set up in triplicate in flask (500 mL) and the experiments lasted for 48 h at 30°C, as described in SI-1 in S1 Text. During mating, the transconjugants were counted and calculated as colony-forming units per milliliter culture (CFU/mL) on Luria-Bertani (LB)-selective plates (containing 100 mg/L of Ap, 60 mg/L of Km, and 10 mg/L of Tc) [19,30]. Periodic sampling (10 mL) was collected at 0, 4, 8, 12, 16, 20, 32, 48 hours and used for plate counting and DNA extraction.

### Plasmid RP4 horizontal transfer microcosms

The counting plate method [32] was used to detect changes of plasmid RP4 horizontal transfer in freshwater microcosms treated with 1.0 g/L of [BMIm][PF6]. The number of total cultivable indigenous recipients (Nr) was determined using culturing on LB agar plates. The number of donor strains (N_4_) (Ap^R^, Km^R^, Tc^R^ and Rif^R^) was enumerated on LB tetra-antibiotic plates containing 60 mg/L Km, 100 mg/L of Ap, 10 mg/L of Tc and 40 mg/L of Rif. Meanwhile, the colonies (N_3_) (Ap^R^, Km^R^ and Tc^R^), which contained donor strains and transconjugants, were counted on LB plates containing antibiotics (containing 100 mg/L of Ap, 60 mg/L of Km, and 10 mg/L of Tc). Furthermore, in parallel, samples from microcosms were plated onto LB tri-antibiotic plates as negative controls to rule out spontaneous mutation of the recipient strains. Horizontal transfer frequency (*f*) was calculated using the formula:

$$f=\left(N_{3}-N_{4}\right)\left(CFU/mL\right)/\left(N_{r}-N_{4}\right)\left(CFU/mL\right).$$ (1)

where *Nr* is the total cultivable indigenous recipients, CFU/mL; *N*_*3*_ is the amount of bacteria carrying RP4 plasmid (Ap^R^, Km^R^ and Tc^R^) containing donors and transconjugants, CFU/mL; and *N*_*4*_ is the donor strains (Ap^R^, Km^R^, Tc^R^ and Rif^R^), CFU/mL. To exclude the possibility that the released plasmid RP4 from the lysed bacterial cells uptake by other recipient bacteria, plasmid RP4 transformation under the same exposure of [BMIm][PF6] was included. The donor *E*.*coli* K12 was grown and microcosm was treated as described earlier. RP4 extraction was performed in *E*.*coli* K12 using a bacterial DNA kit according to the manufacturer’s instructions (Omega, USA). Plasmid RP4 was added to microcosm for final concentrations of 5 μg/mL (this concentration is the equivalent of the donor *E*.*coli* K12 harboring plasmid RP4 at a concentration indicative of conjugative transfer), added to [BMIm][PF6] of at concentrations of 0, 0.001, 0.01, 0.1, 1.0 and 5.0 g/L. For details of these transformations, please refer to SI-6 in S1 Text.

### Identification of transconjugants

Transconjugants were isolated by replica plating [57]. Microcosm samples were plated to tri-antibiotic plates and quad-antibiotic plates by replica plating to insure the same strains are at the same location in these two plates. Transconjugants are those that can grow on tri-antibiotic plates but not on quad-antibiotic plates at the same point. Meanwhile, PCR and DNA sequencing were used to verify that the plasmid RP4 had been transferred into the recipient strains; PCR primers were specially designed and are shown in S2 Table. Transconjugants were incubated in liquid LB tri-antibiotic media containing 100 mg/L of Ap, 50 mg/L of Km, and 10 mg/L of Tc overnight on a shaker incubator (160 rpm) at 37°C. Plasmid DNA was extracted using a plasmid extraction kit (OMEGA, USA) according to the manufacturer’s instructions. Furthermore, the characteristics of transconjugant morphology were recorded, and the isolated transconjugants were also identified by 16S rRNA sequencing.

### Quantification of the *aph*A gene and *tra*F gene

Samples from microcosms (8 mL) were centrifuged and the total DNA extraction was performed using a bacterial DNA Kit according to manufacturer’s instructions (OMEGA, USA). Polymerase chain reaction (PCR) assays were used to target the 16S rRNA gene, *aph*A gene, and *tra*F gene.

Qualitative PCR assays were conducted in a Biometra T100 gradient thermal cycler (Biometra). The PCR mixture (25 μL) contained 1 μL diluted DNA extract as the template, 1.5 μmol/L of each primer (S2 Table), 2 μL 250 μmol/L of each deoxyribonucleoside triphosphate (dNTP), 1.25 U Taq DNA polymerase (TransGen), and 2.5μL of 10xPCR buffer (TransGen). DNA products were diluted from 20 to 50-fold and used in 16S rRNA gene PCR to optimize the template concentration. For each DNA extract, duplicate PCR tubes were analyzed for the presence of each target gene. The negative controls, both the reaction mixture without DNA and the reaction mixture with DNA from the microcosm without the donor were included in each of the PCR run. The PCR procedure for 16S rRNA, *aph*A gene, and *tra*F gene started with an initial DNA denaturation (95°C for 5 min), followed by 30 cycles of 30 sec at 95°C (denaturing), 30 sec of annealing at the temperatures specified in S2 Table, and 1 min at 72°C (extension), followed by a final extension of 7 min at 72°C. The size (S2 Table) and specificity (unique band) of PCR products were determined by comparison with DNA standards (Marker I, TransGen) after 1.5% (w/v) agarose gel electrophoresis [6].

qPCR analyses were performed on a Bio-Rad iQ5 instrument to quantify bacteria 16S rRNA, *aph*A gene, and *tra*F gene. Calibration standard curves for positive controls were generated as previously described. The establishment of negative and positive controls and qPCR reactions were described previously. The primers, amplification details, and standard curves of the 16S rRNA, *aph*A gene, and *tra*F gene are listed in S2 Table.

### Detection of cell membrane permeability effects of [BMIm][PF6]

Flow cytometry (FCM) (BD FACSCalibur) was applied to differentiate and quantify the [BMIm][PF6]-treated group (0.001–5.0 g/L) and control group cells in freshwater microcosms as a function of the percentage of positive cells based on the extent of cell membrane permeability. A higher percentage of positive cells signifies enhanced cell membrane permeability. The FCM was equipped with an excitation wavelength of 488 nm. Propidium iodide (PI) (OMEGA, USA) was used to determine increased membrane permeability cells and control cells, respectively. Microcosm samples (4 mL) were centrifuged and washed 3x with phosphate buffer solution (PBS) (10 g/L NaCl, 0.25 g/L KCl, 1.8 g/L Na_2_HPO4, and 0.3 g/L KH_2_PO_4_). Bacterial cells (the concentration was always less than 10^6^ cells/mL) were then stained with PI (0.01 mg/mL) and incubated in the dark for 8 min before measurement [58]. All data were processed using CellQuest Pro software (USA).

## Results and Discussion

### [BMIm][PF6] promoted the horizontal transfer of plasmid RP4

**Fig 1:**
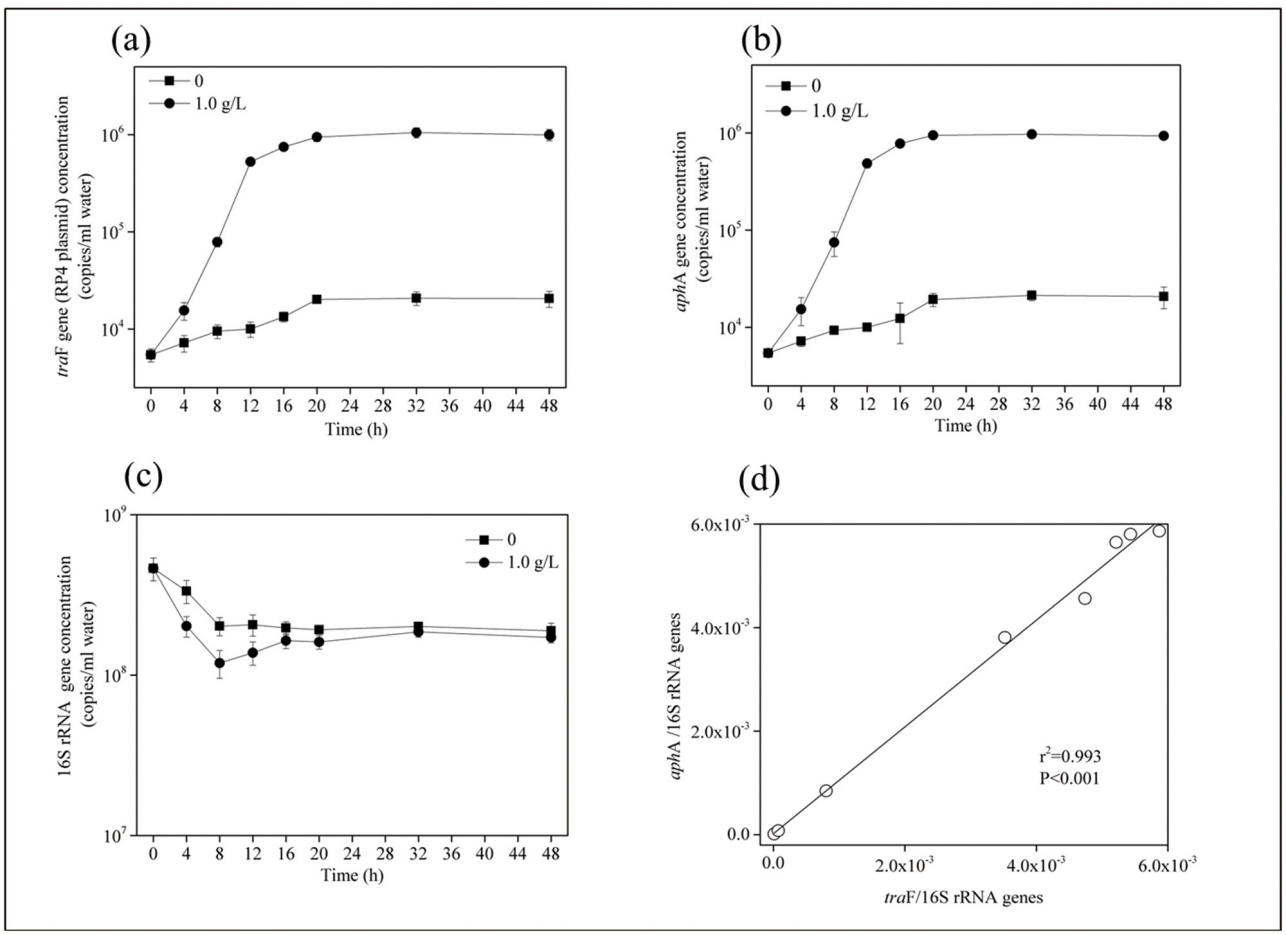
The changes in the *tra*F gene (copies per ml water) (a), *aph*A gene (copies per ml water) (b), and 16S rRNA gene (copies per ml water) levels (c) as well as the correlation between relative abundance of the *aph*A (*aph*A gene /16S rRNA gene) and *tra*F (*tra*F gene /16S rRNA gene) genes (d) in the freshwater microcosms treated with 1.0 g/L [BMIm][PF6].

In the [BMIm][PF6] (1.0 g/L)-treated microcosm, changes of plasmid RP4 abundance were tracked by *tra*F gene (Fig 1a). The abundance of the *tra*F gene increased up to 56-fold higher [(7.48±0.04)×10^5^ copies per mL water] in [BMIm][PF6]-treated groups than [BMIm][PF6] untreated controls [(1.34±0.16)×10^4^ copies per mL water;] (Fig 1a). These facts suggest that [BMIm][PF6] significantly enhanced the proliferation of plasmid RP4 in the microcosm (p < 0.05, S-N-K test).

**Fig 2:**
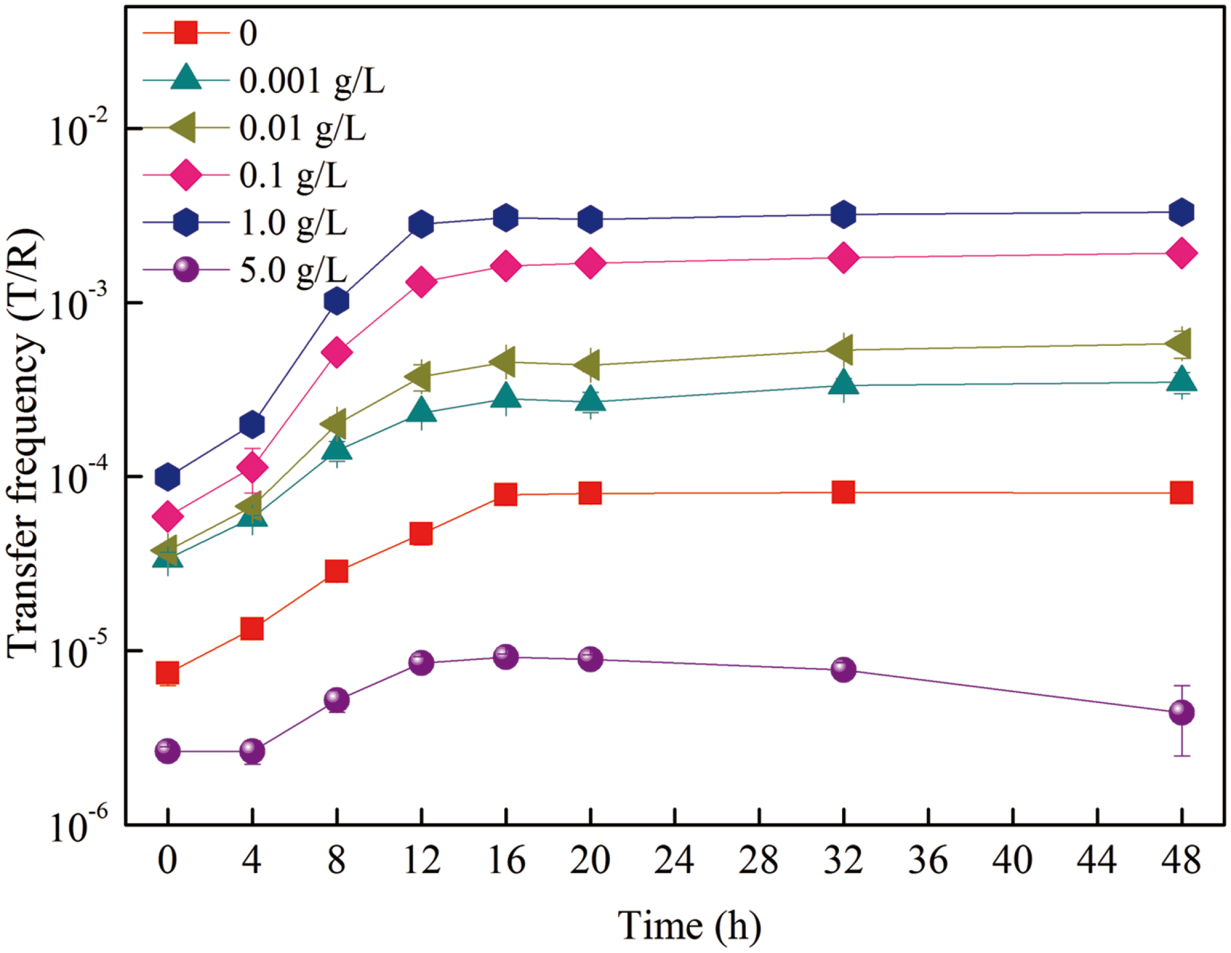
The changes of plasmid RP4 horizontal transfer from *E*.*coli* K12 to indigenous bacteria treated with different concentrations of [BMIm][PF6] in freshwater microcosms.

In the plasmid RP4 horizontal transfer microcosm, the natural transfer frequency of plasmid RP4 in the water environment was very low [30, 59], only (5.10±0.71)×10^-5^ per recipient cell in control groups ([BMIm][PF6] untreated groups) in the present study (Fig 2). This low transfer frequency was similar to those described by previous studies, where the transfer frequency of plasmid RP4 from the *Pseudomonas putida* donor strain to indigenous bacteria in activated sludge was in the range of 4.0×10^-6^ to 1.0×10^-5^ transconjugants per recipient [60]. The presence of an IL [BMIm][PF6] in water significantly promoted the transfer frequency of plasmid RP4 approximately 60-fold [(3.06±0.07)×10^-3^ per recipient cell] higher than [BMIm][PF6] untreated groups (Fig 2). Meanwhile, the transfer frequency increased with increasing concentrations of [BMIm][PF6]). The transfer frequency reached a peak value [(3.06±0.07)×10^-3^ per recipient cell] at 1.0 g/L of [BMIm][PF6] treatment.

**Fig 3:**
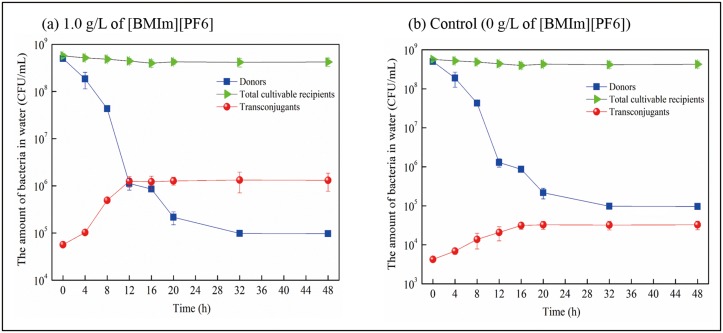
The changes in the number of the donors, the cultivable recipients and the transconjugants during plasmid RP4 horizontal transfer when treated with (a) 1.0 g/L of [BMIm][PF6] and (b) control (0 g/L of [BMIm][PF6]).

For all treatments of [BMIm][PF6], plasmid RP4 horizontal transfer was enhanced and plateaued by 16 hours. In the horizontal transfer microcosms, after inoculation of the donor bacteria, a decrease in cultivated donor bacterial numbers was observed in the initial phase of the horizontal transfer (Fig 3). The donor strains acclimatized to the indigenous bacteria community by decreasing their abundance before stabilizing. In contrast, there was no significant variance (*p* = 0.861) of the growth of cultivable recipients under 1.0 g/L of [BMIm][PF6] exposure during plasmid RP4 horizontal transfer (Fig 3). Meanwhile, the cultivated bacterial number of transconjugants significantly increased and reached the plateau by 16 hours (Fig 3), suggesting the plasmid RP4 horizontal transfer occurred. The increasing transfer frequency of plasmid RP4 is in accordance with the increasing abundance of the *tra*F gene (indicator of plasmid RP4) under [BMIm][PF6] treatment, suggesting that proliferation of plasmid RP4 in the microcosms was mainly caused by horizontal transfer of plasmid RP4, which is promoted by [BMIm][PF6] treatment. Widespread dissemination of ARGs was throughout bacterial populations by vertical transfer and horizontal transfer processes [12]. Acquired resistance occurs between individual cells or species mainly by horizontal transfer [8–10]. In this study, the IL [BMIm][PF6] increased plasmid RP4 transfer frequency is mainly via horizontal transfer from resistant bacteria to indigenous bacteria, which is fundamental for the dissemination of ARGs. Meanwhile, during the gene horizontal transfer process, transconjugant reproduction or replication (defined as the gene vertical transfer) may have simultaneously occurred with horizontal transfer, and this should not be excluded. The calculated of horizontal transfer frequency based on the transconjugants in plasmid mediated horizontal transfer was similar to previous publications [12,23].

Additionally, there was no significant difference (*p* = 0.344) in the abundance of 16S rRNA gene (as a surrogate for total bacteria) between [BMIm][PF6] (1.0 g/L) treated groups and controls (Fig 1c). This result was comparable with previous report that the growth of *E*.*coli*, *P*.*pastoris*, *and B*.*cereus* was unaffected by 1.5 g/L of [BMIM][BF4] and the growth of *P*. *pastoris* was uninhibited by [BMIM][PF6] at 10 g/L [48]. Khudyakov et al. [55] also found that 125 mM (18 g/L) of [C2mim][Cl] had no significant effect on *Enterobacter lignolyticus* strain growth and it can grow in the presence of 500 mM of [C2mim][Cl], illustrating that the IL [C2mim][Cl] as “environmentally friendly” solvents, essentially have non-toxic effect on bacterial growth. Although some ILs under certain concentrations exert neglected effects on bacterial growth both observed in previous studies and our study, due to their remarkable effects on facilitating the horizontal transfer of the plasmid RP4 which is the multi-drug resistance carrier, further consideration on the their application and discharges into the environment should be carefully evaluated.

### Bacterial species identification of transconjugants

**Fig 4:**
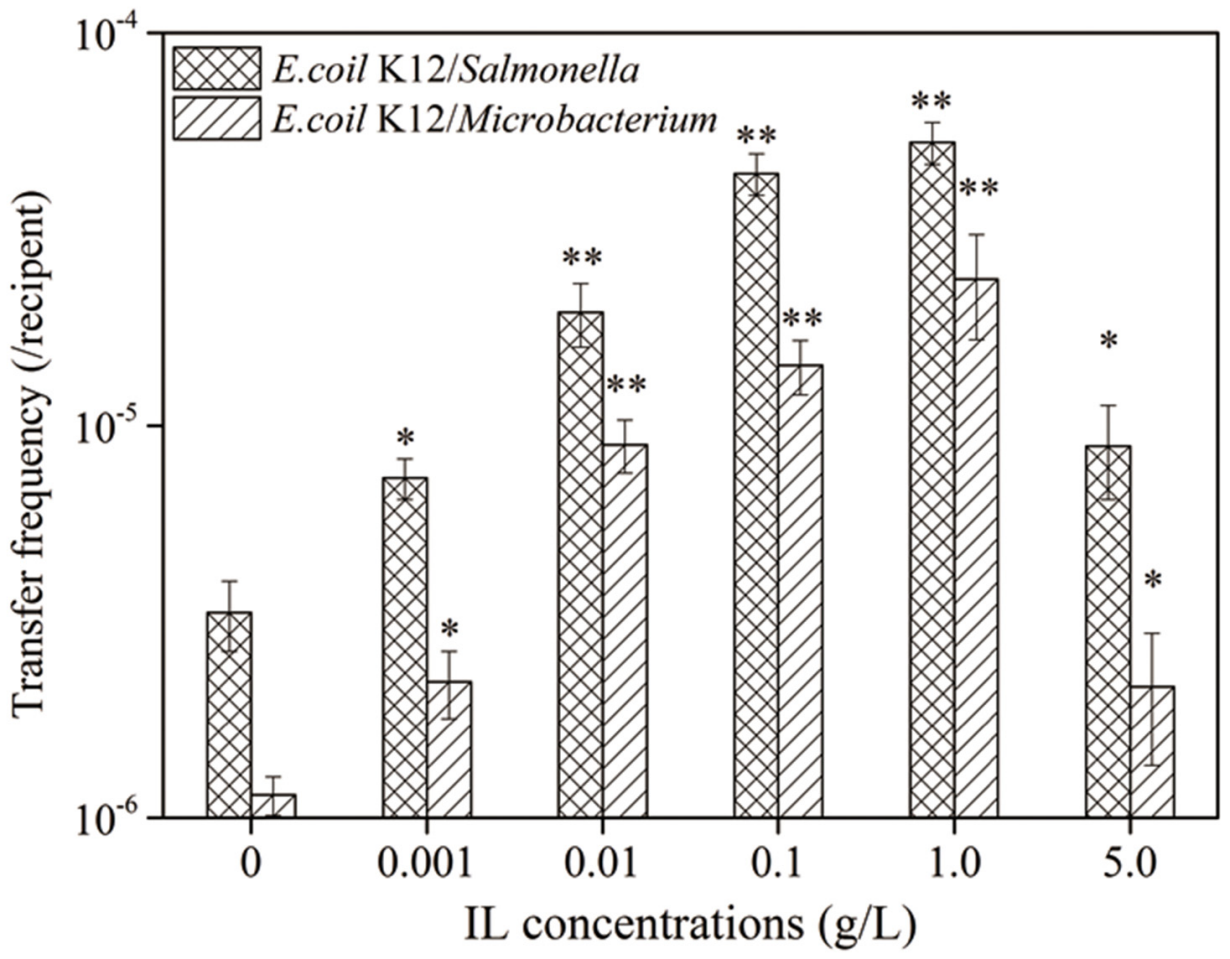
The horizontal transfer of plasmid RP4 from *E*.*coil* K12 to *Salmonella* spp. and from *E*.*coil* K12 to *microbacterium* spp. was affected by [BMIm][PF6] concentration mated for 16 h at pH 7.0 and 30°C. The concentration of [BMIm][PF6] had a significant effect on the transfer frequency of the plasmid RP4 (ANOVA, P < 0.05). Significant differences between the single concentration groups and the control groups (0 g/L group) were tested with the Student-Newman-Keuls (S-N-K) test, *P < 0.05 and **P < 0.01.

Each of the transconjugants was identified by 16S rRNA sequencing and the results showed that the transconjugants belonged to 6 genera and 24 species (Table 1), all of which are indigenous bacteria, indicating that many sensitive indigenous bacterial species in the water environment could possess the resistance plasmid RP4 via horizontal transfer, facilitating the propagation and proliferation of ARGs induced by [BMIm][PF6]. Additionally, most of the cultivable transconjugants (22 of 24 isolated strains) are Gram-negative (Table 1). Compared with the cultivable indigenous bacteria (recipients in microcosm) (S3 Table), Gram-negative bacteria *of Acinetobacter* spp., *Alcaligenes* spp., *Achromobacter* spp. and *Pseudomonas* spp. were identified as dominant plasmid RP4 recipients, as summarized in Table 1. Meanwhile, results of the IL [BMIm][PF6] promoted horizontal transfer of plasmid RP4 from *E*.*coil* K12 to both Gram-negative bacteria *Salmonella* spp. and to Gram-positive bacteria *microbacterium* spp. were shown in Fig 4. The horizontal transfer frequency increased with increasing [BMIm][PF6] concentrations in both recipient as Gram-negative bacteria and Gram-positive bacteria. The horizontal transfer frequency is much higher (3-fold) in recipient of *Salmonella* spp. which is Gram-negative bacteria than *microbacterium* spp. suggesting that Gram-negative bacteria are more likely to acquire ARGs during the horizontal transfer of plasmid RP4, resulting in many indigenous bacteria have to be resistant to several antibiotics simultaneously and capable of transferring their resistance among environmental microorganisms of different genera [61, 62]. This increases the threat of Gram-negative bacteria to public health and poses challenges for developing a new generation of antibiotics against them [13,63]. Particularly notable, two strains of opportunistic pathogen *Acinetobacter* spp. and *Salmonella* spp. were both Gram-negative bacteria and they were isolated among the transconjugants, increasing the risk of ARGs dissemination to human pathogens and posing great threat to public health.

**Table 1:** The identification of cultivable transconjugants.

Genus	Strains	G^+^/ G^−^ *
*Acinetobacter*	8	G^−^
*Alcaligenes*	4	G^−^
*Achromobacter*	4	G^−^
*Pseudomonas*	4	G^−^
*Salmonella*	2	G^−^
*Microbacterium*	2	G^+^

*G^+^, Gram-positive bacteria; G^−^, Gram-negative bacteria.

### [BMIm][PF6]-enhanced cell membrane permeability

**Fig 5:**
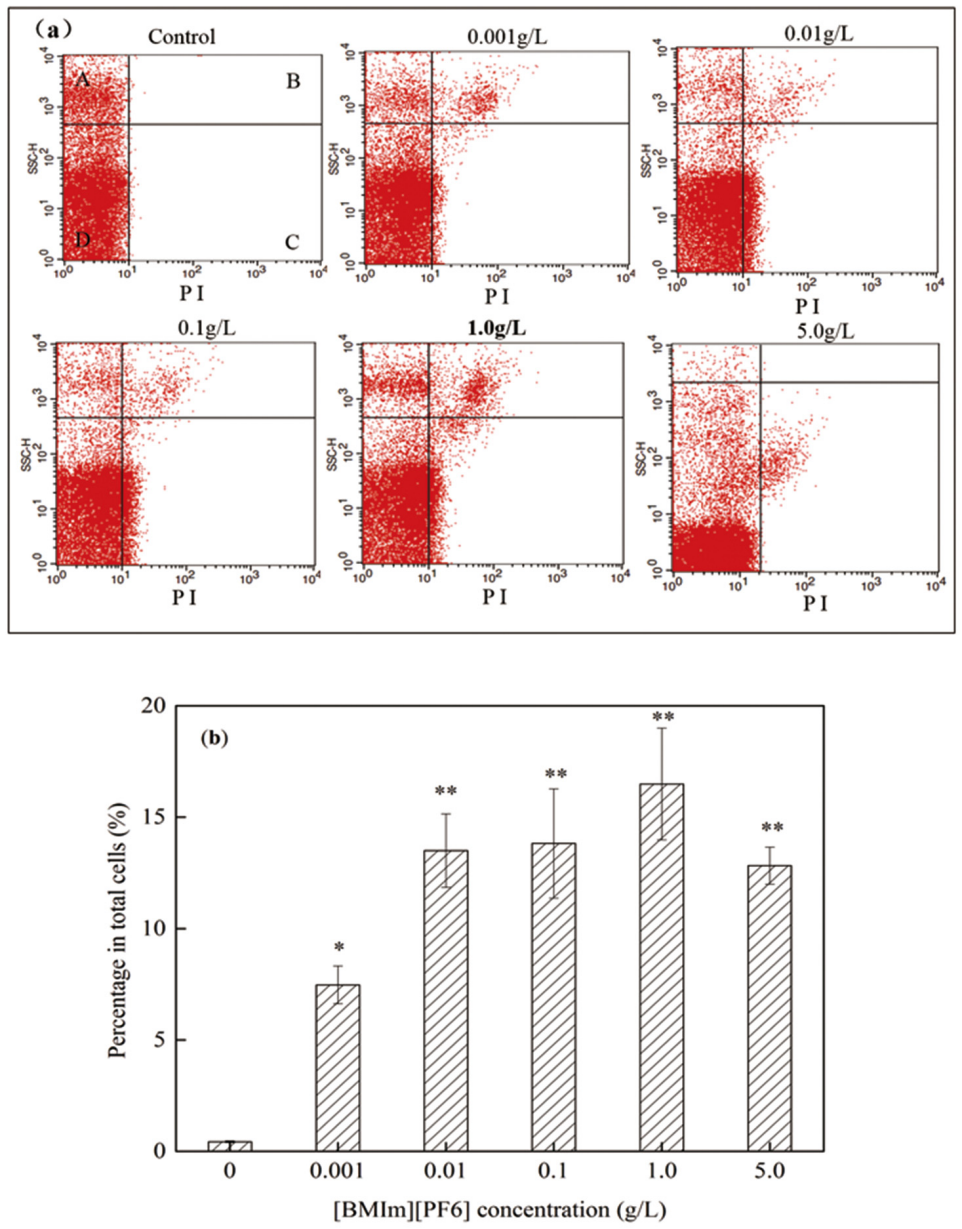
Cell membrane permeability of microorganisms in the freshwater microcosms treated with [BMIm][PF6] quantified using flow cytometry (FCM). (a) Flow cytometry data in a dot plot: Quadrants A and D: negative signal (normal cells); Quadrants B and C: PI-positive (cells with increased cell membrane permeability). (b) Percentage of cells with increased cell membrane permeability (PI positive) in the freshwater microcosms treated with [BMIm][PF6]. [BMIm][PF6] concentration had a significant effect on cell membrane permeability of microorganisms (ANOVA, P < 0.05). Significant differences between single concentration groups and the control groups (0 g/L group) were tested with the Student-Newman-Keuls (S-N-K) test, *P < 0.05 and **P < 0.01. Data points represent the mean ±standard error (n = 3).

FCM assay showed that cell membrane permeability of bacteria treated with [BMIm][PF6] (Fig 5a, quadrant B and C) were significantly increased up to 38-fold higher (16.49%) in 1.0 g/L [BMIm][PF6]-treated groups than [BMIm][PF6] untreated controls (0.43%) (*p*<0.05, S-N-K test) (Fig 5b). The percentage of PI-positive cells (cells with increased cell membrane permeability) increased with increasing concentrations of [BMIm][PF6] treatment (0.001–1.0 g/L) (Fig 5b), indicating that bacterial cell membrane permeability increases with increasing concentrations of [BMIm][PF6] (0.001–1.0 g/L). Meanwhile, transformation was not observed when the donor plasmid RP4 concentration (5 μg/mL) was equivalent to the donor plasmid RP4 concentration of conjugative transfer in [BMIm][PF6]-treated groups and untreated groups. It is therefore reasonable to conclude that [BMIm][PF6] exerted selective pressure promoting the spread of plasmid RP4 by conjugation but not by transformation. The cell membrane serves as barrier to cell permeation and acts as a substantial barrier against penetration [19,64], preventing any compounds (e.g., antibiotics, nutrients, environmental pollutants and genetic exchange) from entering the cell, maintaining the physiological equilibrium [65]. The bacterial cell membranes constitute an important barrier for horizontal transfer of genetic material among bacteria of different species or genera [19]. Bacteria adapt their permeability by modulating the expression of outer membrane porin proteins (OMPs), which are known to play important roles in the membrane transport and to control the permeability of the cellular membrane [66,67]. Pore-forming with modulation of porin proteins allow donor pilus to attach to and access the recipient cell [68,69], facilitating cell-to-cell contact and the conjugative transfer of plasmid RP4. Meanwhile, enhanced cell membrane permeability facilitate communication between recipients and the outer environment, particularly donors, which in turn promote gene transfer of the resistance plasmid [70]. Other chemicals, such as antibiotics [53, 55] and nanomaterials [23] are capable of inducing oxidative stress which is a general adaptation of the organism in exposure of chemicals that can damage cell membranes and may promote the horizontal transfer of genes [71]. The [BMIm][PF6], as chemical stressor and it is possible that they induce stress responses in the bacteria and therefore enhanced cell membrane permeability. Previous studies have reported fluidity of bacterial cell membrane and interference with membrane-bound enzyme activity with exposure to some ILs [48, 55, 72]. This study showed that [BMIm][PF6] suppresses the cell membrane barrier possibly by enhanced cell membrane permeability that is likely to facilitate the transfer of ARGs between indigenous bacteria.

### [BMIm][PF6] enhanced propagation of ARGs mediated by plasmid RP4

Plasmids, as the main vector for resistance genes and carrying multiple resistance genes, play a critical role in horizontal transfer and contribute to the spread and dissemination of ARGs in the environment [24]. In the present study, changes of the *aph*A gene (kanamycin resistance gene which is located on plasmid RP4) followed a similar trend as those of the *tra*F gene (indicator for the abundance of plasmid RP4) (Fig 1b). A positive correlation (*p*<0.01) was found between relative abundance of the *aph*A (*aph*A gene/16S rRNA gene) and *tra*F (*tra*F gene/16S rRNA gene) genes (Fig 1d), implying that the proliferation and propagation of *aph*A gene could be attributed to [BMIm][PF6]-promoted plasmid RP4 horizontal transfer. This study illustrated that [BMIm][PF6] increased ARGs propagation mediated by plasmid RP4 in the water environment. To date, there have been no reports of ILs discharged into the environment; however, the ever increasing production and applications of ILs may accelerate ILs entering the environment and make a variety of bacterial community exposed to ILs, which increased the risk of antibiotic resistance dissemination [46]. The results of this study suggest that careful evaluation is required before bulk emission of some ILs into the aquatic environment.

## Conclusions

This study illustrates that the IL [BMIm][PF6] facilitated the horizontal transfer of ARGs mediated by plasmid RP4 among indigenous bacteria in aquatic environment. Meanwhile, in the horizontal transfer microcosms, two strains of opportunistic pathogen *Acinetobacter* spp. and *Salmonella* spp. were isolated among the transconjugants, illustrating plasmid RP4 mediated horizontal transfer of ARGs occurred in pathogen. This could increase the risk of ARGs dissemination to human pathogens and pose great threat to public health. [BMIm][PF6] compromises the cell membrane barrier by enhanced cell membrane permeability which is likely the mechanism of increased horizontal transfer of plasmid RP4. This study implies that ILs facilitate horizontal transfer of the ARGs, posing great risks to public health. It is suggested that application of ILs in industrial processes should be carefully evaluated before their bulk emission into the aquatic environment.

## Supporting Information

S1 Table The properties of water quality in samples. (DOCX)

S2 Table PCR primers and PCR conditions. (DOCX)

S3 Table The cultivable indigenous bacteria (recipients in microcosm) in freshwater isolated by LB media. (DOCX)

S1 Text Detailed impormation about the horizontal transfer experiment, PCR and qPCR, and water quality analysis. (DOCX)

## References

[1] Aminov RI (2009) The role of antibiotics and antibiotic resistance in nature. Environ Microbiol 11:2970–2988. 10.1111/j.1462-2920.2009.01972.x 19601960

[2] Pruden A, Larsson DG, Amezquita A, Collignon P, Brandt KK, Graham DW, et al (2013) Management options for reducing the release of antibiotics and antibiotic resistance genes to the environment. Environ Health Perspect 121:878–885. 10.1289/ehp.1206446 23735422 PMC3734499

[3] Marti E, Jofre J, Luis Balcazar J (2013) Prevalence of Antibiotic Resistance Genes and Bacterial Community Composition in a River Influenced by a Wastewater Treatment Plant. PLoS ONE 8:e78906 10.1371/journal.pone.0078906 24205347 PMC3808343

[4] Su JQ, Wei B, Xu CY, Qiao M, Zhu YG (2014) Functional metagenomic characterization of antibiotic resistance genes in agricultural soils from China. Environ Int 65:9–15. 10.1016/j.envint.2013.12.010 24412260

[5] Pei R, Kim SC, Carlson KH, Pruden A (2006) Effect of river landscape on the sediment concentrations of antibiotics and corresponding antibiotic resistance genes (ARG). Water Res 40:2427–2435. 16753197 10.1016/j.watres.2006.04.017

[6] Luo Y, Mao D, Rysz M, Zhou Q, Zhang H, Xu L, et al (2010) Trends in antibiotic resistance genes occurrence in the Haihe River, China. Environ Sci Technol 44:7220–7225. 10.1021/es100233w 20509603

[7] Zhang T, Zhang M, Zhang X, Fang HH (2009) Tetracycline resistance genes and tetracycline resistant lactose-fermenting Enterobacteriaceae in activated sludge of sewage treatment plants. Environ Sci Technol 43:3455–3460. 19544839 10.1021/es803309m

[8] Sørensen SJ, Bailey M, Hansen LH, Kroer N, Wuertz S (2005) Studying plasmid horizontal transfer in situ: a critical review. Nat Rev Microbiol 3:700–710. 16138098 10.1038/nrmicro1232

[9] Zhu YG, Johnson TA, Su JQ, Qiao M, Guo GX, Stedtfeld RD, et al (2013) Diverse and abundant antibiotic resistance genes in Chinese swine farms. Proc Natl Acad Sci U S A 110:3435–3440. 10.1073/pnas.1222743110 23401528 PMC3587239

[10] Aminov RI (2011) Horizontal gene exchange in environmental microbiota. Frontmicrobiol 2:1–19. 10.3389/fmicb.2011.00158 PMC3145257 21845185

[11] Alekshun MN, Levy SB (2007) Molecular mechanisms of antibacterial multidrug resistance. Cell 128:1037–1050. 17382878 10.1016/j.cell.2007.03.004

[12] Dodd MC (2012) Potential impacts of disinfection processes on elimination and deactivation of antibiotic resistance genes during water and wastewater treatment. J Environ Monit 14:1754–1771. 10.1039/c2em00006g 22572858

[13] Van Meervenne E, Van Coillie E, Kerckhof FM, Devlieghere F, Herman L, De Gelder LSP, et al (2012) Strain-specific transfer of antibiotic resistance from an environmental plasmid to foodborne pathogens. BioMed Res Int 2012:1–8. 10.1155/2012/834598 PMC3392033 22791963

[14] Chen B, Yang Y, Liang X, Yu K, Zhang T, Li X (2014) Metagenomic Profiles of Antibiotic Resistance Genes (ARGs) between Human Impacted Estuary and Deep Ocean Sediments. Environ Sci Technol 7:12753–12760. 10.1021/es403818e 24125531

[15] Zhang T, Zhang XX, Ye L (2011) Plasmid Metagenome Reveals High Levels of Antibiotic Resistance Genes and Mobile Genetic Elements in Activated Sludge. PLoS One 6: e26041 10.1371/journal.pone.0026041 22016806 PMC3189950

[16] Berglund B, Khan GA, Lindberg R, Fick J, Lindgren PE (2014) Abundance and Dynamics of Antibiotic Resistance Genes and Integrons in Lake Sediment Microcosms. PLoS ONE 9: e108151 10.1371/journal.pone.0108151 25247418 PMC4172728

[17] Cheng W, Chen H, Su C, Yan S (2013) Abundance and persistence of antibiotic resistance genes in livestock farms: A comprehensive investigation in eastern China. Environ Int 61:1–7. 10.1016/j.envint.2013.08.023 24091253

[18] Burch TR, Sadowsky MJ, LaPara TM (2014) Fate of Antibiotic Resistance Genes and Class 1 Integrons in Soil Microcosms Following the Application of Treated Residual Municipal Wastewater Solids. Environ Sci Technol 48:5620–5627. 10.1021/es501098g 24762092

[19] Thomas CM, Nielsen KM (2005) Mechanisms of, and barriers to, horizontal gene transfer between bacteria. Nat Rev Microbiol 3:711–721. 16138099 10.1038/nrmicro1234

[20] Baker-Austin C, Wright MS, Stepanauskas R, McArthur JV (2006) Co-selection of antibiotic and metal resistance. Trends Microbiol 14:176–182. 16537105 10.1016/j.tim.2006.02.006

[21] Knapp CW, McCluskey SM, Singh BK, Campbell CD, Hudson G, Graham DW (2011) Antibiotic Resistance Gene Abundances Correlate with Metal and Geochemical Conditions in Archived Scottish Soils. PLoS ONE 6: e27300 10.1371/journal.pone.0027300 22096547 PMC3212566

[22] Alonso A, Sanchez P, Martinez JL (2001) Environmental selection of antibiotic resistance genes. Environ Microbiol Rep 3:1–9. 10.1046/j.1462-2920.2001.00161.x 11225718

[23] Qiu ZG, Yu YM, Chen Z, Jin M, Yang D, Zhao Z, et al (2012) Nanoalumina promotes the horizontal transfer of multiresistance genes mediated by plasmids across genera. Proc Natl Acad Sci U S A 109:4944–4949. 10.1073/pnas.1107254109 22411796 PMC3323979

[24] Hausner M, Wuertz S (1999) High rates of conjugation in bacterial biofilms as determined by quantitative in situ analysis. Appl Environ Microbiol 65:3710–3713. 10427070 10.1128/aem.65.8.3710-3713.1999 PMC91555

[25] Levy SB, Marshall B (2004) Antibacterial resistance worldwide: causes, challenges and responses. Nat med 10:S122–129. 15577930 10.1038/nm1145

[26] Henschke RB, Schmidt FR (1990) Plasmid mobilization from genetically engineered bacteria to members of the indigenous soil microflora in situ. Curr Microbiol 20:105–110.

[27] Mispagel H, Gray JT (2005) Antibiotic resistance from wastewater oxidation ponds. Water Environ Res 2996–3002; 16381146 10.2175/106143005x73875

[28] Manceau C, Gardan L, Devaux M (1986) Dynamics of RP4 plasmid transfer between Xanthomonas campestris pv. corylina and Erwinia herbicola in hazelnut tissues, in planta. Canj microbiol 32:835–841.

[29] Johannes Sørensen S, Schyberg T, Rønn R (1999) Predation by protozoa on Escherichia coli K12 in soil and transfer of resistance plasmid RP4 to indigenous bacteria in soil. Appl Soil Ecol 11:79–90.

[30] Inoue D, Sei K, Soda S, Ike M, Fujita M (2005) Potential of predominant activated sludge bacteria as recipients in conjugative plasmid transfer. J biosci bioeng 100:600–605. 16473767 10.1263/jbb.100.600

[31] Sorensen SJ (1993) Transfer of plasmid RP4 from Escherichia coli K-12 to indigenous bacteria of seawater. Microbial releases:viruses, bacteria, fungi. 2:135–141. 8111533

[32] Soda S, Otsuki H, Inoue D, Tsutsui H, Sei K, Ike M (2008) Transfer of antibiotic multiresistant plasmid RP4 from escherichia coli to activated sludge bacteria. J Biosci Bioeng 106:292–296. 10.1263/jbb.106.292 18930008

[33] Earle MJ, Seddon KR (2000) Ionic liquids. Green solvents for the future. Pure Appl Chem 72:1391–1398.

[34] Rogers RD, Seddon KR (2003) Ionic liquids—solvents of the future? Science 302:792–793. 14593156 10.1126/science.1090313

[35] Olivier H (1999) Recent developments in the use of non-aqueous ionic liquids for two-phase catalysis. J Mol Catal A-Chem 146:285–289.

[36] Wishart JF (2009) Energy applications of ionic liquids. Energ& Environ Sci 2:956–961. 10.1038/srep07206 25425458 PMC4244623

[37] Domínguez de María P, Maugeri Z (2011) Ionic liquids in biotransformations: from proof-of-concept to emerging deep-eutectic-solvents. Curr opin chem biol 15:220–225. 10.1016/j.cbpa.2010.11.008 21112808

[38] Roosen C, Müller P, Greiner L (2008) Ionic liquids in biotechnology: applications and perspectives for biotransformations. Appl Microbiol Biot 81:607–614. 10.1007/s00253-008-1730-9 18979095 PMC7419490

[39] Liu J, Jiang G, Jönsson JÅ. (2005) Application of ionic liquids in analytical chemistry. TrAC-Trend Anal Chem 24:20–27.

[40] Welton T (1999) Room-temperature ionic liquids. Solvents for synthesis and catalysis. Chem Rev 99:2071–2084. 11849019 10.1021/cr980032t

[41] Plechkova NV, Seddon KR (2008) Applications of ionic liquids in the chemical industry. Chem Soc Rev 37:123–150. 10.1039/b006677j 18197338

[42] Kubisa P (2005) Ionic liquids in the synthesis and modification of polymers. J PolymSciPol Chem 43:4675–4683.

[43] Lu J, Yan F, Texter J (2009) Advanced applications of ionic liquids in polymer science. ProgPolym Sci 34:431–448.

[44] Antonietti M, Kuang DB, Smarsly B, Yong Z (2004) Ionic liquids for the convenient synthesis of functional nanoparticles and other inorganic nanostructures. Angew ChemInt Edit 43:4988–4992. 15372641 10.1002/anie.200460091

[45] Li Z, Jia Z, Luan Y, Mu T (2008) Ionic liquids for synthesis of inorganic nanomaterials. Curr Opin Solid St M 12:1–8.

[46] Markiewicz M, Jungnickel C, Arp HPH (2013) Ionic Liquid Assisted Dissolution of Dissolved Organic Matter and PAHs from Soil Below the Critical Micelle Concentration. Environ Sci Technol 47:6951–6958. 10.1021/es304568w 23627900

[47] Stepnowski P (2005) Solid-phase extraction of room-temperature imidazolium ionic liquids from aqueous environmental samples. Anal Bioanal Chem 381:189–193. 15616779 10.1007/s00216-004-2932-3

[48] Ganske F, Bornscheuer UT (2006) Growth of Escherichia coli, Pichia pastoris and Bacillus cereus in the presence of the ionic liquids [BMIM][BF4] and [BMIM][PF6] and organic solvents. Biotechnol lett 28:465–469. 16614927 10.1007/s10529-006-0006-7

[49] Romero A, Santos A, Tojo J, Rodriguez A (2008) Toxicity and biodegradability of imidazolium ionic liquids. J Hazard Mater 151:268–273. 18063302 10.1016/j.jhazmat.2007.10.079

[50] Kelman D, Kashman Y, Rosenberg E, Ilan M, Ifrach I, Loya Y (2001) Antimicrobial activity of the reef sponge Amphimedon viridis from the Red Sea: evidence for selective toxicity. Aquat Microb Ecol 24:9–16.

[51] Li G, Shen J, Zhu Y (1998) Study of pyridinium‐type functional polymers. II. Antibacterial activity of soluble pyridinium‐type polymers. J Appl Polym Sci Liu 67:1761–1768.

[52] Nancharaiah YV, Reddy GKK, Lalithamanasa P, Venugopalan VP (2012) The ionic liquid 1-alkyl-3-methylimidazolium demonstrates comparable antimicrobial and antibiofilm behavior to a cationic surfactant. Biofouling 28:1141–1149. 10.1080/08927014.2012.736966 23092364

[53] Docherty KM, Kulpa CF Jr (2005) Toxicity and antimicrobial activity of imidazolium and pyridinium ionic liquids. Green Chem 7:185–189.

[54] Ranke J, Mölter K, Stock F, Bottin-Weber U, Poczobutt J, Hoffmann J, et al (2004) Biological effects of imidazolium ionic liquids with varying chain lengths in acute Vibrio fischeri and WST-1 cell viability assays. Ecotox environ safe 58:396–404. 10.1016/S0147-6513(03)00105-2 15223265

[55] Khudyakov JI, D'Haeseleer P, Borglin SE, Deangelis KM, Woo H, Lindquist EA, et al (2012) Global transcriptome response to ionic liquid by a tropical rain forest soil bacterium, Enterobacter lignolyticus. Proc Natl Acad Sci U S A 109:2173–2182. 10.1073/pnas.1112750109 PMC3420158 22586090

[56] OECD (2002) Aerobic and Anaerobic Transformation in Aquatic Sediment Systems In Guidelines for Testing of Chemicals No 308. QECD.

[57] Lederberg J, Lederberg EM (1952) Replica plating and indirect selection of bacterial mutants. J Bacteriol 63:399–406. 14927572 10.1128/jb.63.3.399-406.1952 PMC169282

[58] Xue Z, Sendamangalam VR, Gruden CL, Seo Y (2012) Multiple roles of extracellular polymeric substances on resistance of biofilm and detached clusters. Environ Sci Technol 46:13212–13219. 10.1021/es3031165 23167565

[59] Daane L, Molina J, Sadowsky M (1997) Plasmid transfer between spatially separated donor and recipient bacteria in earthworm-containing soil microcosms. Appl Environ Microbiol 63:679–686. 16535521 10.1128/aem.63.2.679-686.1997 PMC1389527

[60] Geisenberger O, Ammendola A, Christensen BB, Molin S, Schleifer KH, Eberl L (1999) Monitoring the conjugal transfer of plasmid RP4 in activated sludge and in situ identification of the transconjugants. FEMS Microbiol Lett 174:9–17. 10234817 10.1111/j.1574-6968.1999.tb13543.x

[61] Colomer-Lluch M, Calero-Caceres W, Jebri S, Hmaied F, Muniesa M, Jofre J (2014) Antibiotic resistance genes in bacterial and bacteriophage fractions of Tunisian and Spanish wastewaters as markers to compare the antibiotic resistance patterns in each population. Environ Int 73:167–175. 10.1016/j.envint.2014.07.003 25127043

[62] Agerso Y, Petersen A (2007) The tetracycline resistance determinant Tet 39 and the sulphonamide resistance gene sulII are common among resistant Acinetobacter spp. isolated from integrated fish farms in Thailand. J Antimicrob Chemother 59:23–27. 17095527 10.1093/jac/dkl419

[63] Kumarasamy KK, Toleman MA, Walsh TR, Bagaria J, Butt F, Balakrishnan R, et al (2010) Emergence of a new antibiotic resistance mechanism in India, Pakistan, and the UK: a molecular, biological, and epidemiological study. Lancet Infect Dis 10:597–602. 10.1016/S1473-3099(10)70143-2 20705517 PMC2933358

[64] Nikaido H (1988) Bacterial resistance to antibiotics as a function of outer membrane permeability. JAntimicrob Chemoth 22:17–22. 10.1093/jac/22.supplement_a.17 3062001

[65] Lavigne JP, Sotto A, Nicolas‐Chanoine MH, Bouziges N, Bourg G, Davin‐Regli A (2012) Membrane permeability, a pivotal function involved in antibiotic resistance and virulence in Enterobacter aerogenes clinical isolates. Clin Microbiol Infec 18:539–545. 10.1111/j.1469-0691.2011.03607.x 21883663

[66] Osborn MJ, Wu HC (1980) Proteins of the outer membrane of gram-negative bacteria. Annual review of microbiology 34: 369–422. 6254441 10.1146/annurev.mi.34.100180.002101

[67] Ozkanca R, Sahin N, Isik K, Kariptas E, Flint KP (2002) The effect of toluidine blue on the survival, dormancy and outer membrane porin proteins (OmpC and OmpF) of Salmonella typhimurium LT2 in seawater. Journal of applied microbiology 92:1097–1104. 12010550 10.1046/j.1365-2672.2002.01642.x

[68] Achtman M, Morelli G, Schwuchow S (1978) Cell-cell interactions in conjugating Escherichia coli: role of F pili and fate of mating aggregates. Journal of bacteriology 135:1053–61. 357413 10.1128/jb.135.3.1053-1061.1978 PMC222482

[69] Guglielmini J, Neron B, Abby SS, Garcillan-Barcia MP, de la Cruz F, Rocha EPC (2014) Key components of the eight classes of type IV secretion systems involved in bacterial conjugation or protein secretion. Nucleic Acids Research 42:5715–5727. 10.1093/nar/gku194 24623814 PMC4027160

[70] Achouak W, Heulin T, Pages JM (2001) Multiple facets of bacterial porins. FEMS microbiology letters 199:1–7. 11356559 10.1111/j.1574-6968.2001.tb10642.x

[71] Beaber JW, Hochhut B, Waldor MK (2004) SOS response promotes horizontal dis-semination of antibiotic resistance genes. Nature 427:72–74. 14688795 10.1038/nature02241

[72] Pernak J, Sobaszkiewicz K, Mirska I (2003) Anti-microbial activities of ionic liquids. Green Chem 5:52–56.

