# Supplementary material for: Enhanced Horizontal Transfer of Antibiotic Resistance Genes in Freshwater Microcosms Induced by an Ionic Liquid
Source: PLoS One. 2015 May 7;10(5):e0126784. doi: 10.1371/journal.pone.0126784 (PMC4423773; doi:10.1371/journal.pone.0126784)
Supplement: S1 Table — (DOCX) [file pone.0126784.s001.docx]

**Table S1.** Freshwater properties of samples collected from the Water Parke in Tianjin, China

|  | Measurements |
| --- | --- |
| Enrofloxacin (ng/L) | 36.0 |
| Tetracycline (ng/L) | 300.4 |
| Sulfamethazine (ng/L) | 0.4^*^ |
| Ciprofloxacin (ng/L) | 26.8 |
| Ofloxacin (ng/L)  kanamycin | 10.0  N.D^**^ |
| Oxytetracycline (ng/L) | 11.6 |
| Sulfamethoxazole (ng/L) | 82.4 |
| Doxycycline (ng/L)  Streptomycin (ng/L) | 5.2  50.5 |
| Chlortetracycline (ng/L) | 16.8 |
| Roxithromycin (ng/L)  Ampicillin  Rifampaaicin | 7.2  N D  N D |
| Zinc (ug/L) | 50.0 |
| Copper (ug/L)  TOC (mg/L)  NH3-N (mg/L)  Dissolved P (mg/L) | 10.0  16.8  3.2  0.2 |
| pH | 7.2 |
| Water temperature (°C) | 30 |

^*^ >The limit of detection (LOD); ^**^ ND, not detected.
