# Supplementary material for: Enhanced Horizontal Transfer of Antibiotic Resistance Genes in Freshwater Microcosms Induced by an Ionic Liquid
Source: PLoS One. 2015 May 7;10(5):e0126784. doi: 10.1371/journal.pone.0126784 (PMC4423773; doi:10.1371/journal.pone.0126784)
Supplement: S2 Table — (DOCX) [file pone.0126784.s002.docx]

**Table S2.** PCR primers and PCR conditions

| Primer | Target | Sequence (5’-3’) | PCR annealing temp (ºC) | qPCR annealing temp (ºC) | Amplicon size (bp) | Source |
| --- | --- | --- | --- | --- | --- | --- |
| 16s-FW | *16S*  *rRNA* | CGGTGAATACGTTCYCGG | 58 | 57.5 | 126 | [1] |
| 16s-RV |  | GGWTACCTTGTTACGACTT |  |  |  |  |
| 27F | *16S*  *rRNA* | AGAGTTTGATCCTGGCTCAG | 56 | - | 1466 | [2] |
| 1492R |  | GGTTACCTTGTTACGACTT |  |  |  |  |
| *aph*A-FW | *aph*A | GGCTTCGTGATGCCTGCTT | 62 | 62 | 198 | This study |
| *aph*A-RV |  | CATTCCTGGCCGTGGTTCT |  |  |  |  |
| *traF*-FW | *traF* | CTCCGATGGAGGCCGGTAT | 54.1 | 54.1 | 196 | This study |
| *traF*-RV |  | GGGAATGCCATCTGCCTTGA |  |  |  |  |

*FW, forward; RV, reverse.

**REFERENCES**

1. Suzuki MT, Taylor LT, DeLong EF (2000) Quantitative analysis of small-subunit rRNA genes in mixed microbial populations via 5′-nuclease assays. Appl Environ Microbiol 66:4605-4614.

2. Lane D (1999) 16S/23S rRNA sequencing. Nucleic acid techniques in bacterial systematics.
