# Supplementary material for: Enhanced Horizontal Transfer of Antibiotic Resistance Genes in Freshwater Microcosms Induced by an Ionic Liquid
Source: PLoS One. 2015 May 7;10(5):e0126784. doi: 10.1371/journal.pone.0126784 (PMC4423773; doi:10.1371/journal.pone.0126784)
Supplement: S3 Table — (DOCX) [file pone.0126784.s003.docx]

**Table S3.** The cultivable indigenous bacteria (recipients in microcosm) in freshwater isolated by LB media.

| Genus | G^＋^/ G^－^* |
| --- | --- |
| *Acinetobacter* | G^－^ |
| *Alcaligenes* | G^－^ |
| *Achromobacter* | G^－^ |
| *Pseudomonas*  *Plesiomonas* | G^－^  G^－^ |
| *Salmonella*  *Bacillus* | G^－^  G^＋^ |
| *Microbacterium*  *Staphylococcus*  *Streptococcus*  *Rhodococcus* | G^＋^  G^＋^  G^＋^  G^＋^ |

*G^＋^, Gram-positive bacteria; G^－^, Gram-negative bacteria.
