# Supplementary material for: Enhanced Horizontal Transfer of Antibiotic Resistance Genes in Freshwater Microcosms Induced by an Ionic Liquid
Source: PLoS One. 2015 May 7;10(5):e0126784. doi: 10.1371/journal.pone.0126784 (PMC4423773; doi:10.1371/journal.pone.0126784)
Supplement: S1 Text — (DOCX) [file pone.0126784.s004.docx]

**Supplementary Information for**

**Enhanced horizontal transfer of antibiotic resistance genes in freshwater microcosms induced by** **an ionic liquid**

Qing Wang ^1^, Daqing Mao ^2*^, Quanhua Mu ^1^, Yi Luo ^1*^

**^1^** College of EnvironmenPtal Science and Engineering, Ministry of Education Key Laboratory of Pollution Processes and Environmental Criteria, Nankai University, Tianjin 300071, China.

**^2^** School of Environmental Science and Engineering, Tianjin University, Tianjin 300072, China.

1. Horizontal transfer experiment in freshwater microcosms

Microcosm horizontal transfer experiments, based on the OECD 308 test [1], were established to determine the effect of Ionic Liquid [BMIm][PF6] on the plasmid RP4 from *E.coli* K12 (ATCC 47076) on indigenous bacteria in freshwater microcosms. The freshwater sample was collected from the Water Parke in Tianjin, China on Sep 2013. Water properties were described in Table S1. The strain of rifampicin resistance (Rif^R^) *E.coli* K12 harboring the plasmid RP4, carrying ampicillin, kanamycin and tetracycline resistance (Ap^R^, Km^R^, and Tc^R^), was used as the RP4 donor. The recipients were indigenous bacteria in freshwater microcosms that tested negative both for the donor strain and the bacteria carrying the RP4 plasmid.

The water samples were stabilized for 3 hours at 4°C in a refrigerator to remove sediment. Supernatant (200 mL) was collected and supplemented with 1% (V:V) Luria-Bertani (LB) media, incubated overnight on a shaker incubator (160 rpm) at 30°C and diluted to reach microbial concentrations (the optical densities) of OD_600_=0.400. Samples were then supplemented with 1% (V:V) *E.coli* K12 donor strains, making freshwater microcosms.

The microcosm horizontal transfer experiments were set up in a 500 mL flask. Microcosms were spiked with IL [BMIm][PF6] (purity>99%, Chinese Academy of Science, China), resulting in initial freshwater concentrations of 0, 0.001, 0.01,0.1, 1.0, and 5.0 g/L, according to previous publications [2-5], and mixed by vortexing.

The microcosm horizontal transfer experiments were stabilized in a climate cabinet at 30°C for 48 h. 10 mL of microcosm samples were sampled at 0, 4, 8, 12, 16, 20, 32, 48 hours and used for plate counting and DNA extraction.

2. PCR and qPCR conditions of target genes

Qualitative PCR assays were conducted in a Biometra T100 gradient (Biometra, Germany) as follows (25 μL): The PCR procedure for an initial DNA denaturation (95°C for 5 min), followed by 30 cycles of 30 sec at 95°C (denaturing), 30 sec of annealing at the temperatures specified in Table S2, and 1 min at 72°C (extension), followed by a final extension of 7 min at 72°C.

qPCR analyses were performed on a Bio-Rad iQ5 instrument (Bio-Rad Company, CA, USA). Amplification (25 μL) consisted of an initial cycle of initial denaturing 5 min at 95℃, followed by 40 cycles of 15 s at 95℃, 1 min at annealing temperatures (S2 Table), 30 s at 72℃ and a final melt curve stage with temperature ramping from 55℃ to 95℃(0.5℃ per read, 30 s hold) [6].

3. Standard curves

Standard curves were established before qPCR analyses on a Bio-Rad iQ5 instrument (Bio-Rad Company, CA, USA) to quantify 16S rRNA, *aph*A and *TraF* genes. After normal PCR amplification, fresh PCR products of 16S rRNA, *aph*A, and *TraF* genes were recovered, purified and ligated onto a pEASY-T3 vector (TransGen, China) and transformed to *E.coli* DH5α according to manufacturer’s instructions (TransGen, China). Positive clones were screened by blue-white selection, and PCR and sequencing were used to verify cloning of the target genes. Plasmids carrying target genes were chosen as the standards for qPCR. Plasmids carrying target genes were extracted according to the plasmid extraction kit (OMEGA, USA). The concentration and quality of the plasmid were determined by spectrophotometric analysis and agarose gel electrophoresis. The copy number of target genes per microliter of plasmid solution was calculated as previously described [7]. Eight-point standard curves for qPCR were generated using 10-fold serial dilutions of the plasmid carrying target genes. R^2^ values were higher than 0.990 for all standard curves. According to the standard curves, the Ct value of samples was used to calculate the gene copies of 16S rRNA, *aph*A, and *TraF* genes.

4. Statistical analysis

Data analysis was conducted using SPSS Statistics Version 20.0 (IBM, NY, USA). The correlation between relative abundance of the *aph*A (*aph*A gene /16SrRNA gene) and *tra*F (*tra*F gene /16SrRNA gene) genes (Fig. 1d) was analyzed using a Pearson correlation analysis. One-way ANOVA was conducted to analyze the results between group samples with SPSS Statistics Version 20.0 (IBM, NY, USA). The averages and standard deviations of the data were calculated using Excel 2007 (Microsoft Inc., USA). The cell membrane permeability of microorganisms induced by [BMIm][PF6] concentration in the freshwater microcosms (Fig. 4b) was conducted using the Student-Newman-Keuls (S-N-K) test. The cut-off level for statistical significance was *p* < 0.05. OriginPro 9.0 (Origin Lab Corporation, USA) was used for plotting.

5. The properties of water quality in samples

Analysis of antibiotics was conducted using HPLC-MS/MS, and details including Sample pretreatment, solid phase extraction and HPLC MS/MS Analysis are described in our previous study [6]. Samples were collected into sterile, aluminum-covered containers (to prevent phaotodegradation of antibiotics) and store d at 4°C during transport to the laboratory, where water samples were stored at 4°C until sample pretreatment, which occurred within 24 h of the sample collection.

The recovery (%), the limit of quantification (LOQ) and reproducibility of response (peak area relative standard deviation (RSD), expressed as % of the mean) were used to validate of antibiotics detection method. In this study, The recovery (%) from water samples ranged from 70 to 95 for sulfonamides, 63 to 80aaa for quinolones, 62 to 81 for tetracyclines, and 59~80 for macrolide. Recoveries of (Trimethyl-^13^C_3_ caffeine, meclocycline, sulfadiazine-^13^C6 and lomefloxacin, as surrogate to compensate for matrix effects during water pretreatment) for water samples was 71%~90%. The limit of quantification (LOQ) was determined as the lowest concentration which generated a signal to noise ratio (S/N) ≥ 10. The LOQ for the 14 antibiotics under consideration ranged from 1.5 ng/L to 3.2 for water. The RSD of the 14 compounds ranged from 0.5% to 2.5% for water.

Heavy metals were quantified using inductively coupled plasma mass spectrometry (ICP-MS) (Sciex Elan DRC-e, USA).

TOC analysis used a TOC analyzer (analytikjena multi N/C 3100, Germany), NH3-N analysis used ammonium-Nessler’s reagent spectrophotometry. Dissolved phosphorus (dP) was analyzed by Mo-Sb antispectrophotometry method.

1. The plasmid RP4 transformation system

To rule out transformation from the naked DNA of the donor to the recipient bacteria in the conjugative transfer process, transformation experiments [8] were used as negative controls of transfer from naked DNA (plasmid RP4) to recipients (indigenous bacteria in microcosm) treated with [BMIm][PF6]. The donor *E.coli* K12 was grown and microcosm was treated as described earlier.

RP4 extraction was performed in *E.coli* K12 using a bacterial DNA kit according to the manufacturer’s instructions (Omega, USA). Donor plasmid RP4 was added to microcosm for final concentrations of 5 µg/mL (this concentration is the equivalent of the donor *E.coli* K12 harboring plasmid RP4 at a concentration indicative of conjugative transfer), added to [BMIm][PF6] of at concentrations of 0, 0.001, 0.01, 0.1, 1.0 and 5.0 g/L, and mixed.

Transformation experiments were performed in a climate cabinet at 30 °C and the experimental methods used were the same as those used for conjugation. Transformation frequency (*f*) was calculated using the formula: *f* = N_T_ (cfu/mL)/N_r_ (cfu/mL) [N_T_: the number of transconjugants (Ap^R^, Km^R^, Tc^R^); N_r_: the total cultivable indigenous recipients].

The total cultivable indigenous recipients were also plated onto LB plates containing antibiotics (containing 100 mg/L of Ap, 60 mg/L of Km, and 10 mg/L of Tc) as negative controls to rule out the spontaneous mutation of the cultivable indigenous recipients.

**REFERENCES**

1. OECD (2002) Aerobic and Anaerobic Transformation in Aquatic Sediment Systems. In Guidelines for Testing of Chemicals No 308. Organisation for Economic Cooperation and Development.

2. Docherty KM, Kulpa Jr CF (2005) Toxicity and antimicrobial activity of imidazolium and pyridinium ionic liquids. Green Chem 7:185-189.

3. Stepnowski P, Mrozik W, Nichthauser J (2007) Adsorption of alkylimidazolium and alkylpyridinium ionic liquids onto natural soils. Environ Sci Technol 41:511-516.

1. Pham TP, Cho CW, Yun YS (2010) Environmental fate and toxicity of ionic liquids: A review. Water Res 44:352-372.

5. Wells AS, Coombe VT (2006) On the freshwater ecotoxicity and biodegradation properties of some common ionic liquids. Organic Process Research & Development 10:794-798.

6. Luo Y, Mao D, Rysz M, Zhou Q, Zhang H, et al. (2010) Trends in antibiotic resistance genes occurrence in the Haihe River, China. Environ Sci Technol 44:7220-7225.

7. Zhang T, Zhang M, Zhang X, Fang HH (2009) Tetracycline resistance genes and tetracycline resistant lactose-fermenting Enterobacteriaceae in activated sludge of sewage treatment plants. Environ Sci Technol 43:3455-3460.

8. Overballe-Petersen S, Harms K, Orlando L A A, Mayar J V M, Rasmussen, Set al. (2013) Bacterial natural transformation by highly fragmented and damaged DNA. Proc Natl Acad Sci U S A 110:19860-19865.
